# Intra-cavity stem cell therapy inhibits tumor progression in a novel murine model of medulloblastoma surgical resection

**DOI:** 10.1371/journal.pone.0198596

**Published:** 2018-07-10

**Authors:** Onyinyechukwu Okolie, David M. Irvin, Juli R. Bago, Kevin Sheets, Andrew Satterlee, Abigail G. Carey-Ewend, Vivien Lettry, Raluca Dumitru, Scott Elton, Matthew G. Ewend, C. Ryan Miller, Shawn D. Hingtgen

**Affiliations:** 1 Division of Pharmacoengineering and Molecular Pharmaceutics, UNC Eshelman School of Pharmacy, University of North Carolina at Chapel Hill, Chapel Hill, North Carolina, United States of America; 2 Lineberger Comprehensive Cancer Center, University of North Carolina at Chapel Hill, Chapel Hill, North Carolina, United States of America; 3 Division of Neuropathology, Department of Pathology and Laboratory Medicine, School of Medicine, University of North Carolina at Chapel Hill, Chapel Hill, North Carolina, United States of America; 4 Department of Neurology, School of Medicine, University of North Carolina at Chapel Hill, Chapel Hill, North Carolina, United States of America; 5 Neuroscience Center, School of Medicine, University of North Carolina at Chapel Hill, Chapel Hill, North Carolina, United States of America; 6 UNC Human Pluripotent Stem Cell Core, Genetics Department, UNC School of Medicine, The University of North Carolina at Chapel Hill, Chapel Hill, North Carolina, United States of America; 7 Department of Neurosurgery, University of North Carolina at Chapel Hill, Chapel Hill, North Carolina, United States of America; 8 UNC Neuroscience Center, School of Medicine, The University of North Carolina at Chapel Hill, Chapel Hill, North Carolina, United States of America; 9 Biomedical Research Imaging Center, University of North Carolina at Chapel Hill, Chapel Hill, North Carolina, United States of America; University of Pécs Medical School, HUNGARY

## Abstract

**Background:**

Cytotoxic neural stem cells (NSCs) have emerged as a promising treatment for Medulloblastoma (MB), the most common malignant primary pediatric brain tumor. The lack of accurate pre-clinical models incorporating surgical resection and tumor recurrence limits advancement in post-surgical MB treatments. Using cell lines from two of the 5 distinct MB molecular sub-groups, in this study, we developed an image-guided mouse model of MB surgical resection and investigate intra-cavity NSC therapy for post-operative MB.

**Methods:**

Using D283 and Daoy human MB cells engineered to express multi-modality optical reporters, we created the first image-guided resection model of orthotopic MB. Brain-derived NSCs and novel induced NSCs (iNSCs) generated from pediatric skin were engineered to express the pro-drug/enzyme therapy thymidine kinase/ganciclovir, seeded into the post-operative cavity, and used to investigate intra-cavity therapy for post-surgical MB.

**Results:**

We found that surgery reduced MB volumes by 92%, and the rate of post-operative MB regrowth increased 3-fold compared to pre-resection growth. Real-time imaging showed NSCs rapidly homed to MB, migrating 1.6-fold faster and 2-fold farther in the presence of tumors, and co-localized with MB present in the contra-lateral hemisphere. Seeding of cytotoxic NSCs into the post-operative surgical cavity decreased MB volumes 15-fold and extended median survival 133%. As an initial step towards novel autologous therapy in human MB patients, we found skin-derived iNSCs homed to MB cells, while intra-cavity iNSC therapy suppressed post-surgical tumor growth and prolonged survival of MB-bearing mice by 123%.

**Conclusions:**

We report a novel image-guided model of MB resection/recurrence and provide new evidence of cytotoxic NSCs/iNSCs delivered into the surgical cavity effectively target residual MB foci.

## Introduction

Medulloblastoma (MB) is the most common primary brain tumor in children [1, 2]. Molecular analysis has now shown that MB can be sub-divided into 5 molecular subtypes, with distinct transcriptional and epigenetic signatures. Standard MB treatment consists of maximal surgical resection followed by radiation and adjuvant multi-drug chemotherapy [3, 4]. This treatment yields a 5-year survival rate of 60–70% [5], but the nature of these treatments causes damage to the developing brain, and often leaves survivors suffering long-term neurological and developmental defects.[6] In the set of children for which MB remains fatal, the highly aggressive nature of MB cells allows the cancer to evade surgical resection and escape chemo-radiation treatment [7, 8]. There is a significant need to develop new therapies to target the residual MB cells that remain after surgery, without the adverse effects on the non-diseased developing brain caused by current treatment strategies. Developing accurate pre-clinical models to test these therapies will be critical to ensure these new treatment strategies are efficacious in eventual patient testing.

Engineered neural stem cells (NSCs) are emerging as a promising strategy for treating cancer [9–12]. NSCs display inherent tumor tropism and migrate toward distant and invasive intracranial tumor foci including; malignant gliomas, metastases from systemic cancers, and MB [13–17]. Additionally, NSCs can be engineered to deliver a variety of therapeutic agents directly into primary and invasive brain tumors, significantly reducing solid tumor volumes and extending the survival of tumor-bearing mice [9, 15, 16, 18–20]. Although these studies suggest NSC therapy could be highly effective in MB treatment, the lack of pre-clinical models accurately mimicking MB surgical resection limits the advancement of NSC therapy into clinical patient testing [21–23]. Previously, we found surgical tumor removal caused genetic, molecular, and pathologic changes, which modify the post-operative tumor into a fundamentally different disease than the pre-operative solid neoplasm [24], and had profound effects on the delivery and efficacy of stem cell therapies [18, 20, 25]. This suggests studying of the persistence, fate, and migration of NSCs within the MB surgical cavity, as well as the efficacy of cytotoxic NSC therapies against the residual MB that remains after surgery, is critical to advancing this approach to human patient testing and requires the development of an accurate pre-clinical MB model of resection in mice.

Here, we utilized human MB cell lines to create the first mouse model of image-guided MB resection and recurrence. We paired this model with both traditional and novel NSC types to explore multiple aspects of intra-cavity NSC therapy as a new approach to MB treatment. Real-time intra-operative optical imaging allowed resection of 92% of MB volumes. We found post-operative MB exhibited 3-fold faster growth rates compared to pre-operative MB, and observed complete recurrence of the tumor within 5 days post-surgery. Despite the highly aggressive nature of the post-operative cancer, cytotoxic NSCs seeded in the surgical cavity markedly suppressed growth of residual MB volumes and more than doubled the survival of tumor-bearing mice. As a novel approach to personalized therapy in patients, human induced NSCs derived from the skin of pediatric patients (hp-iNSCs) were found to migrate to MB, deliver clinically relevant therapies to slow post-surgical MB recurrence, and prolong median survival. These findings provide the first evidence that intra-cavity NSC therapy is an effective treatment for post-operative MB, and serve as a foundation for advancing this approach towards human patient testing.

## Materials and methods

### Cell culture

Human MB (DAOY, D283) and mouse NSC (C17.2) lines were cultured at 37°C and 5% CO_2_ in complete media consisting of DMEM (Gibco, Grand Island, NY) supplemented with 10% heat-inactivated fetal bovine serum (Sigma, St. Louis, MO), and penicillin/streptomycin (100 μg/mL, Sigma), as previously described.[24] hp-iNSCs were generated using Sox2 and feeder-free conditions as described previously and cultured in STEMdiff Neural Induction Medium (StemCell Technologies, Vancouver, Canada) containing doxycycline (10 μg/ml, Sigma).[15]

### Orthotopic xenografts and fluorescence-guided microsurgery

Daoy-Green Fluorescent Protein / Firefly Luciferase (GFPFL) or D283-GFPFL xenografts were established in the right posterior fossa of Nude mice. A craniotomy aided in the visualization of the underlying GFP+ tumor for image-guided tumor resection. Established xenografts were excised using surgical dissection and aspiration methods similar to those previously described[26]. Bioluminescent imaging (BLI) was used to measure tumor burden pre-resection, immediately post-resection, and at serial time points post-surgery to determine growth rate of recurrent tumors. Animal studies were approved by the University of North Carolina Institutional Animal Care and Use Committee. IACUC protocol #16–082.

### SC migration to human medulloblastoma *in vitro* and *in vivo*

NSCs (NSC-mCherry-FLuc (mcF) or hp-iNSC-mcF) were seeded in the presence or absence of MB cells. Cell populations were separately seeded into adjacent wells located 0.5 mm apart in two-chamber culture-inserts (Ibidi, Verona, WI), placed in glass bottom microwell dishes (MatTek, Ashland, MA), and incubated overnight in complete media. Cells were placed in a VivaView incubator microscope (Olympus) and imaged at 10X every 20 minutes for 24 h.

To assess the *in vivo* migratory capability, NSCs were injected into the contralateral cerebellar hemisphere of mice bearing established Daoy xenografts. Mouse brains were harvested three weeks after tumor cell implantation and processed as described[24].

### *In vitro* and *in vivo* therapy with survival analysis

To assess the *in vitro* therapeutic efficacy of thymidine kinase-expressing stem cell therapy, NSCs or human pediatric induced neural stem cells (hp-iNSC^tk^) were cultured with D283-GFPFL or Daoy-GFPFL, respectively in black walled 96-well plates. Ganciclovir (GCV) was added to the wells at 20 μg/ml and incubated for 72 hr. Media was then aspirated and replaced with 7.5 mg/ml luciferin. After allowing 10 minutes for substrate to react with the enzyme active in living cells, the IVIS kinetic was used to capture bioluminescent signal emanating from surviving cancer cells.

To assess the *in vivo* therapeutic efficacy of thymidine kinase-expressing stem cell therapy, hp-iNSC^tk^ or NSC^tk^ were implanted into the surgical cavity following microsurgical tumor debulking as previously described.[18] Twenty-four hours following surgery, mice received systemic administration of the prodrug GCV (100 mg/kg) or saline. BLI were captured and analyzed as previously described to measure tumor burden and evaluate therapeutic response^[^^26^^]^. Mice were monitored and sacrificed upon the development of neurological symptoms.

## Results

### Creating an image-guided model of orthotopic human MB resection and recurrence in mice

**Fig 1:**
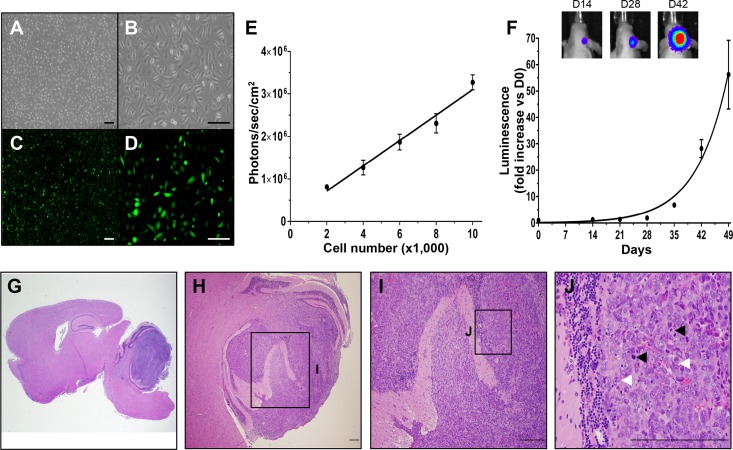
Daoy human MB cells engineered to express GFP and firefly luciferase form orthotopic xenografts in vivo. Cultured Daoy human MB cells transduced with lentiviral vectors encoding GFP-FLuc (Daoy-GFPFL) express GFP and luciferase in vitro as determined by white light (A, B) and fluorescence imaging (C, D). Daoy-GFPFL cell number showed linear correlation with Fluc activity (R^2^ = 0.978, P = 0.001, E). Daoy-GFPFL xenografts showed exponential growth with a doubling time of 5.6 days *in vivo* measured by bioluminescence imaging. Representative bioluminescence images are shown for days 14, 24 and 42 after injection of Daoy-GFPFL cells (F). Representative hematoxylin and eosin staining (G-J) of brain sections show large intra-cerebellar tumors 63 days after injection of Daoy-GFPFL cells. Tumors showed histopathological features of MB, including frequent cell wrapping (white arrowheads) and heightened level of mitosis (black arrowheads). Original magnifications: 15X (G), 40X (A, C), 100X (B, D), 200X (H), 400X (I), 600X (J). Scale bars, 100 μm (A-D) and 200 μm (G-J). n = 5.

Surgical resection remains a component of the clinical standard of care for MB patients, yet preclinical MB models utilize solid MB models and fail to incorporate surgical resection and subsequent MB regrowth. We developed the first orthotopic image-guided MB resection/recurrence model to test NSC-based therapies against the post-surgical disease. Daoy human MB cells were transduced with a lentiviral vector encoding a bi-modal fusion between GFP and firefly luciferase (Daoy-GFPFL; Fig 1A, Fig 1B, Fig 1C, Fig 1D). *In vitro* fluorescence imaging confirmed efficient transduction and robust GFP expression (Fig 1C, Fig 1D). Dilution assays showed the luciferase activity of the Daoy-GFPFL was linearly correlated with cell number (Fig 1E). When Daoy-GFPFL were implanted into the cerebellum of mice to establish orthotopic xenografts, serial bioluminescence images (BLI) showed gradual tumor growth (doubling time 5.6 days), reaching 59-fold at day 49 post-implant (Fig 1F). Histological analysis of post-mortem brain tissue confirmed the presence of large solid tumors in the cerebellum (Fig 1G). The tumors demonstrated hallmarks of MB (Fig 1H, Fig 1J), including frequent cell wrapping (Fig 1J; white arrowheads) and mitotic division (Fig 1J; black arrowheads).

**Fig 2:**
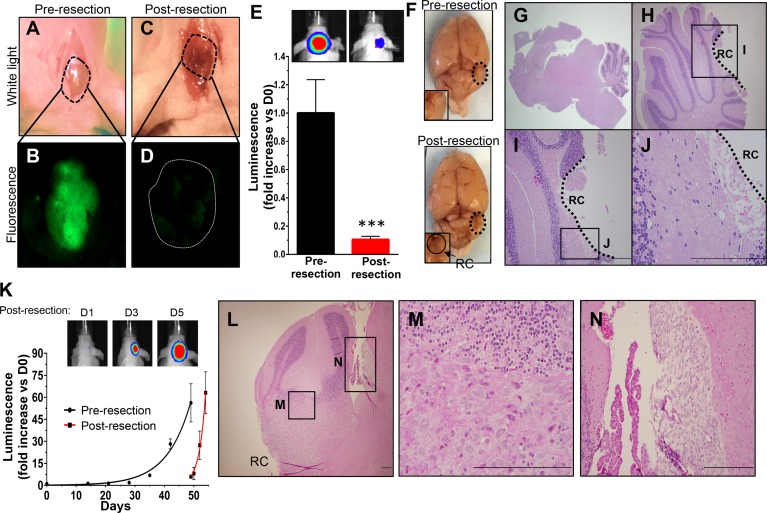
Fluorescence-guided microsurgical resection reduces volumes of orthotopic Daoy-GFPFL xenografts that redevelop. A scalp incision was made 49 days after injection of Daoy-GFPFL cells and craniotomy (A). Fluorescence was used to visualize the underlying GFP^+^ tumors pre resection (B). Fluorescence-guided microsurgery significantly reduced tumor burden as determined by intra-operative imaging (C, D). Post-operative bioluminescence imaging showed a >92% reduction in mean tumor burden (***P = 0.009, E). Representative white light and bioluminescence images pre- and post-resection are shown (E,F). Representative H&E images (G-J) of brain sections taken immediately post-resection did not show apparent residual tumor within the resection cavity. Recurrent Daoy-GFPFL xenografts (K) grew faster than pre-resection counterparts (data from Fig 1F), with doubling times of 1.5 vs. 5.6 days, respectively (P = 0.0003). Representative BLI images are shown for days 1, 3, and 5 post-surgery. H&E images (L-N) of brain sections shows recurrent tumor near the resection cavity and cancer cells disseminating through the cerebral spinal fluid (CSF). Original magnifications: 15X (G), 200X (L, H), 400X (I), 600X (J, M, N). Scale bars, 200 μm. RC, Resection Cavity. n = 5 in each group.

After MB foci were established, craniotomies were performed to expose the underlying neoplasm (Fig 2A and 2B). The tumor was then resected using real-time intraoperative fluorescence imaging to guide the surgery. White light (Fig 2A, Fig 2C) and fluorescence (Fig 2B, Fig 2D) images confirmed efficient debulking of the tumor. Comparison of pre- and post-resection BLI demonstrated surgical reduction of the MB tumor volumes by 92% (Fig 2E). Post-mortem visualization of gross brain tissue confirmed generation of a surgical cavity, while histological analysis verified efficient tumor removal with the majority of tumor burden near the resection cavity no longer present (Fig 2F, Fig 2G, Fig 2H, Fig 2I, Fig 2J).

In contrast to the gradual growth of pre-resection tumors, serial BLI showed MB rapidly re-developed following resection. Post-operative Daoy-GFPFL tumors grew significantly faster than the pre-resection counterparts, with doubling times of 1.5 days compared to 5.6 days, respectively (P = 0.0003; Fig 2K). Histological analysis confirmed MB re-developed 5 days after surgery (Fig 2L). Similar to clinical findings [27], the re-developed tumors spread throughout the cerebellum (Fig 2L, Fig 2M) and disseminated into the CSF (Fig 2N). These features allow this model to improve the pre-clinical study of MB, as it mimics tumor growth, allows for surgical resection, and simulates growth characteristics of recurrent MB in the clinical setting.

### NSCs migrate towards medulloblastoma cells *in vitro* and *in vivo*

**Fig 3:**
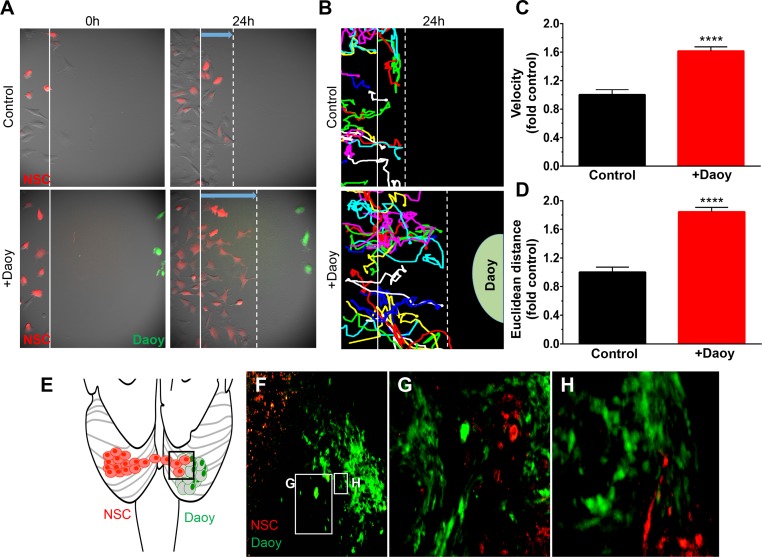
NSCs migrate towards human MB in vitro and in vivo. Migration of NSC-mcF cells (red) the presence (+Daoy) or absence (control) of MB cells (Daoy-GFPFL cells, green) was monitored over 24h (A). Time-lapse images were captured every 20 mins and used to construct single cell tracings (B). Quantitative analysis of single cell tracings demonstrated the presence of Daoy-GFPFL increases the migratory velocity of NSCs (1.61-fold; ****P<0.0001, C), and euclidean distance traveled (1.8-fold; P<0.0001, D). Illustration of NSC and Daoy-GFPFL cells implanted in opposite cerebellar hemispheres (E). Fluorescent images (F-H) 21 days after stem cell implantation shows NSCs co-localize with Daoy-GFPFL tumor cells. Original magnifications: 100X (A, B,), 200X (F, G). n = 4 in panel E.

Stem cells have emerged as powerful tools in brain cancer therapy, as the tumor-homing capacity allows these cells to populate distant tumor foci [15, 18, 19, 28, 29]. We performed real-time motion analysis of NSCs co-cultured in the presence or absence of Daoy MB cells to study the tumor-homing abilities of NSCs in the context of pediatric brain cancer. NSCs randomly migrated when cultured alone, but migrated directly and rapidly toward Daoy MB cells seeded 500μm away as shown by time-lapse fluorescence images and corresponding line tracings of single cell movement (Fig 3A, Fig 3B). Quantitative analysis of these tracings revealed that the addition of MB increased the rate of NSC migration by 61% (Fig 3C; ****P <0.0001) and migratory distance by 84% (Fig 3D; ****P <0.0001) when compared to NSC migration in the absence of MB cells. To investigate NSC tumor-homing *in vivo*, we implanted Daoy-GFPFL (green) MB cells into the cerebellum of mice as illustrated in Fig 3E. Twenty-one days later, NSCs (red) were implanted into the opposite cerebellar hemispheres and allowed to migrate for 2 weeks. Immunofluorescence imaging of post mortem tissue sections revealed that NSCs migrated toward the MB cells (Fig 3F). NSCs were distributed along the tumor tissue interface (Fig 3G) and throughout the core of the tumor (Fig 3H).

### Cytotoxic NSCs show therapeutic efficacy *in vitro* and *in vivo*

**Fig 4:**
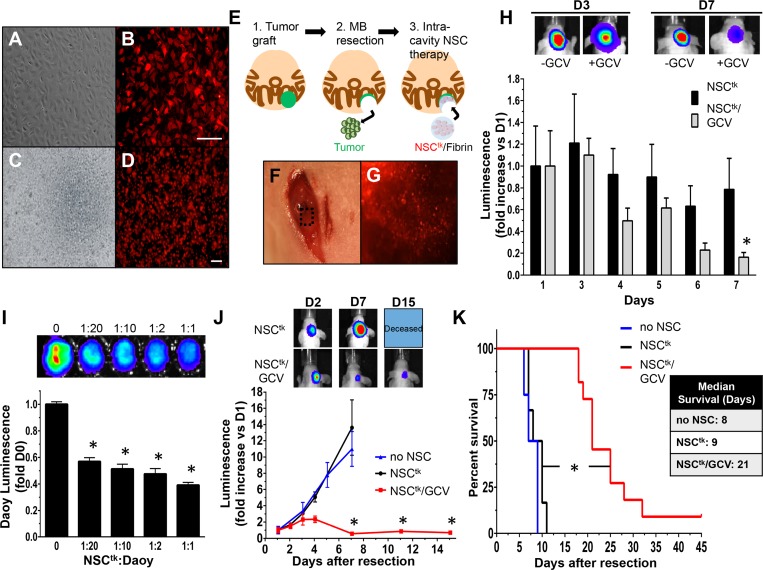
Therapeutic NSCs delivered within the resection cavity regress and delay re-growth of post-surgical MB. White light and fluorescent images of NSC expressing TK (NSC^tk^) in culture (A, B) or mixed with fibrin matrix (NSC^tk^/fibrin) (C, D), illustrate high transduction efficiency and efficient expression of the construct. Depiction of the *in vivo* therapy schema, in which tumors were grafted within the cerebellum, resected and treated with therapeutic NSC^tk^/fibrin (E). Representative white light (F) and fluorescent (G) images confirm NSC^tk^/fibrin implanted within the resection cavity. NSC^tk^/fibrin persist longer in the brain without systemic administration of GCV (NSC^tk^) compared to with systemic administration of GCV (NSC^tk^/GCV) (H). Representative BLI images of NSC^tk^ and NSC^tk^/GCVmice at days 3 and 7 post-surgery are shown above. Daoy-GFPFL cells were seeded with varying concentrations of NSC^tk^ (I). Tumor cell viability was measured by luminescence 24h after GCV administration. Summary graph and representative BLI show a significant reduction of tumor burden in NSC^tk^/fibrin+GCV-treated mice compared to control-treated (J; no NSC and NSC^tk^). NSC^tk^/fibrin with GCV treatment extended median survival two-fold compared to controls (9 vs. 21 days, log rank *P<0.0001, K). Original magnification: 40X (C, D), 100X (A, B). Scale bars = 100 μm. n = 4 per group in the panel H, n = 4 in no NSC and NSC^TK^, n = 7 in the NSC^TK^/GCV group in J-K.

Utilizing our expertise in post-surgical models and intra-cavity therapy for adult brain cancer, we next investigated a new approach to MB therapy where cytotoxic, tumor-homing NSCs are seeded into the tumor cavity to target the aggressive MB cells that remain after surgery. To this end, NSCs were engineered to express the enzyme thymidine kinase (NSC^tk^) that converts the pro-drug ganciclovir (GCV) into a toxic agent leads to induce tumor cell death[30]. White light and fluorescent imaging confirmed high transduction efficiency and efficient expression of the reporter in culture (Fig 4A, Fig 4B) and in the fibrin matrix (Fig 4C, Fig 4D) used to implant the cells in the post-operative surgical cavity. To first explore the persistence of NSC^TK^ within the surgical cavity, the cells were additionally engineered with firefly luciferase. Established MB were then resected from the cerebellum and NSC^tk^ cells were implanted into the surgical cavity in a fibrin matrix (Fig 4E). The NSC^tk^/fibrin matrix was visualized within the surgical cavity with both intra-operative white light (Fig 4F) and fluorescent (Fig 4G) imaging to confirm efficient seeding. Following surgery and implantation of NSC^tk^/fibrin, mice were administered either saline (control) or the GCV pro-drug and serial BLI was used to monitor the fate of NSC^tk^ over 7 days. We found that levels of NSC^tk^ in control mice did not significantly change over a 1 week period, however NSC^tk^ levels in GCV-treated mice decreased 89% over 7 days (Fig 4H). The decrease of NSC^tk^ in the presence of GCV reduces the potential for uncontrolled growth of therapeutic NSCs.

To investigate the NSC^tk^:MB ratio that induces tumor cell death, Daoy-GFPFL cells were seeded *in vitro* with varying dilutions of NSC^tk^. NSC^tk^ induced dose-dependent killing of Daoy-GFPFL cells, with ratios as low as 1:20 leading to a 45% reduction in tumor cell viability (Fig 4I). To investigate the *in vivo* efficacy of NSC^tk^ therapy for post-surgical MB, established Daoy-GFPFL xenografts were surgically resected and NSC^tk^/fibrin were delivered into the resection cavity. Tumors recurred rapidly in untreated mice and mice receiving NSC^tk^/fibrin without GCV. One week after surgery, tumor volumes increased 12-fold in these animals with an average survival time of 8–9 days post-surgery (Fig 4K). In contrast, GCV administration significantly inhibited post-operative tumor progression. Tumor volumes in the NSC^tk^/fibrin + GCV group were 20-fold smaller than control mice 7 days post-treatment (Fig 4J; NSC^tk^/GCV) and median survival more than doubled compared to controls (Fig 4K; 21 vs. 9 days; *P <0.0001). Collectively, these data provide the first evidence of intra-cavity NSC therapy in the treatment of recurrent MB.

**Fig 5:**
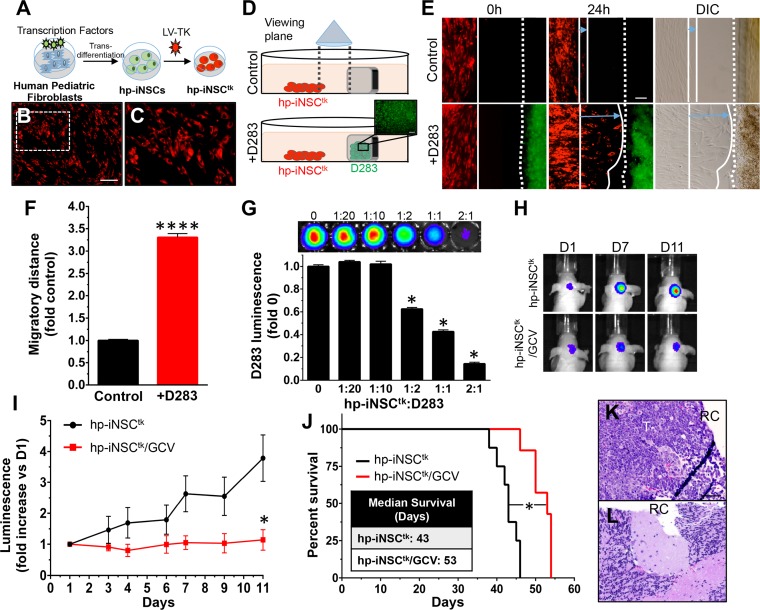
Cytotoxic human pediatric stem cells derived from patient skin suppress and delay regrowth of post-surgical medulloblastoma. Illustration of the reprogramming scheme which converts human pediatric fibroblasts into cytotoxic human pediatric induced neural stem cells (hp-iNSC^tk^, A). hp-iNSC express the thymidine kinase/RFP lentiviral vector as determined by white light (B) and fluorescent (C) images. Schematic depiction of hp-iNSC^tk^ migration assay in the presence (+D283) or absence (control) of MB cells (D). The cells were seeded 500 μm apart from each other and migration was tracked over the course of 24h by fluorescent and white light imaging (E). Summary graph shows hp-iNSC^tk^ cells migrate further in the presence D283-GFPFL cells than in the absence of these cells (3.3-fold, P < 0001, F). D283-GFPFL cells were seeded with varying concentrations of hp-iNSC^tk^ and tumor cell viability was measured by luminescence 24h after GCV administration (G). Representative BLI images (H) and summary graph (I) showing the *in vivo* therapeutic efficacy of cytotoxic NSC therapy (hp-iNSC^tk^/GCV) compared to control (hp-iNSC^tk^). hp-iNSC^tk^/GCV treatment extended survival of mice compared to the control hp-iNSC^tk^ (53 vs 43 days) (J). Post-mortem histopathological analysis showed the presence of larger recurrent tumors in control mice (K) compared with treatment (L) groups. RC and T denotes resection cavity and tumor, respectively. Original magnification: 100X (B, C, E, K, L). Scale bars = 100 μm. n = 5–6 in each group.

Ideally, NSCs would be easily isolated and autologous to avoid immune rejection. Our group previously utilized next-generation direct reprogramming technology to convert skin cells to induced neural stem cells (iNSCs) that migrated to tumors and delivered therapeutic agents that successfully reduced human glioblastoma xenografts in mice. [15, 31] Adaptation of this approach for MB could improve MB therapy in pediatric patients. To investigate iNSC therapy in MB, human pediatric fibroblasts were converted into iNSCs (hp-iNSCs) through forced expression of the neural-specific transcription factor, SOX2, followed by culture in NSC-promoting media [32] (Fig 5A). After conversion, the hp-iNSCs were engineered to express RFP-labeled thymidine kinase (hp-iNSC^tk^). Transduction efficiency was confirmed with fluorescent imaging (Fig 5B, Fig 5C). Using our unique co-culture and kinetic-imaging assays, we found that hp-iNSC^tk^ cells exhibited extensive tropism toward MB cells (Fig 5D, Fig 5E) *in vitro* and migrated over three times further when compared to cells cultured without MB cells (Fig 5F).

MB tumors are comprised of five distinct molecular subgroups; wnt, sonic hedgehog (Shh), group 3, and group 4) [33]. The Daoy cell line falls within the second most common molecular subgroup, Shh. Tumors of this subgroup lead to intermediate prognosis and do not commonly metastasize [34]. As a more stringent test of hp-iNSC^tk^ therapy, we recreated the therapeutic paradigm in a second MB post-surgical model using the more aggressive group 3 tumor cell line D283 [35]. Group 3 tumors frequently metastasize and have the worst prognosis of all 4 molecular subgroups of MB [34]. In *in vitro* co-culture experiments, hp-iNSC^tk^ efficiently killed D283 MB tumor cells (Fig 5G), suggesting *in vivo* potency of hp-iNSC^tk^ therapy against a second molecularly distinct MB cell line. To test hp-iNSC^tk^ therapy *in vivo*, MB tumors were established in the brains of mice using D283 cells expressing GFP and firefly luciferase (D283-GFPFL) and subsequently surgically resected using fluorescence-guided microsurgery. The hp-iNSC^tk^/fibrin cells were delivered into the post-operative resection cavity. Eleven days after treatment initiation, hp-iNSC^tk^/fibrin+GCV was found to suppress tumor recurrence by 332% (Fig 5H, Fig 5I) and extend median survival compared to control mice (Fig 5J; 53 vs. 43 days; *P = 0.0004). Post-mortem histological analysis confirmed the presence of large recurrent tumors in the control groups (Fig 5K) and minimal residual tumors in the hp-iNSC^tk^/fibrin+GCV-treated mice 11 days after treatment (Fig 5L). This model of hp-iNSC^tk^ therapy suggests tumor homing stem cells hold therapeutic potential in patients with MB.

**Fig 6:**
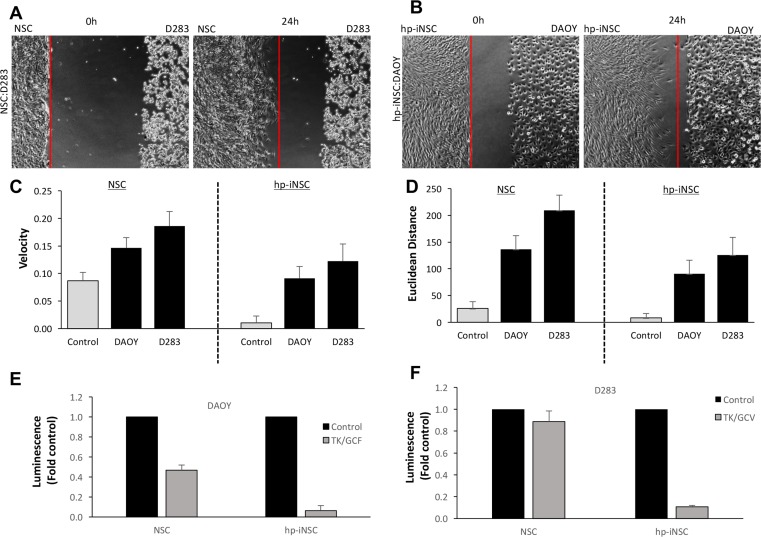
Comparison of NSC and hp-iNSC migration and killing. Representative images showing the migration of NSCs to D283 (A) and hp-iNSC to DAOY cells (B). (C-D) Summary graphs demonstrating the velocity (C) and Euclidean Distance of both NSC and hp-iNSC migrating to DAOY and D283. (E-F) Summary graphs demonstrating the efficacy of NSCTK or hp-iNSCTK therapy for DAOY (E) or D283 (F). Original magnification: 100X (A,B). Scale bars = 100 μm. n = 3–4 in each group.

Having observed that both NSCs and hp-iNSC migrate to MB cells and induce tumor killing, we performed head-to-head comparisons to better understand difference between the two drug carriers. To this end, we first tested the migration of NSC to D283 cells and hp-iNSC to DAOY cells. Real-time kinetic imaging showed both cells types migrated to the MB cells over 24 hrs while minimal migration was detected in control wells (Fig 6A, Fig 6B, S1 Fig). Quantitative analysis revealed that NSCs displayed a faster migration velocity and covered a great Euclidean distance than iNSCs for both MB types (Fig 6C, Fig 6D). However, both the velocity and Euclidean distance in control wells was greater for NSCs than hp-iNSC, suggesting the cells may have inherently greater promiscuity. We next explored the ability of each cell type to kill MB cells. NSC^tk^ or hp-iNSC^tk^ were co-cultured with both DAOY and D283. Viability assays showed that both NSC drug carriers again induced robust killing of both MB cell lines. However, we found that hp-iNSC^tk^ induced greater killing compared to the NSC^tk^ in both MB cell lines (Fig 6E, Fig 6F). Together, these results begin to show differences between NSCs and emerging skin-derived hp-iNSC drug carriers.

## Discussion

Medulloblastoma (MB) is the most common childhood brain malignancy with a peak incidence at 7 years of age.[1] Despite technological advances in the standard of care, nearly all patients who survive experience debilitating side-effects.[2, 3, 36] Thus, there remains an urgent need to minimize the life-changing side effects of treatment by providing alternative therapies.[5] In this study, we developed a new MB resection and recurrence model to determine the impact of surgery on tumor recurrence and evaluate cytotoxic NSCs as a potential therapeutic approach. Using this model, we show that intracavity NSC therapy is effective against MB and may provide a method to control the post-operative disease in humans.

Although surgical resection is a universal component of the standard care for MB patients, surgery remains the least studied component of MB therapy. The events following surgical resection of MB have not been well explored, but recent studies using glioma models have reported increased tumor aggressiveness after surgical resection.[20, 26] In this study, we revealed that recurrent MB tumors exhibited a three-fold increase in proliferation after surgery (Fig 2). Our model is the first image-guided orthotopic model of MB resection and recurrence. Furthermore, our findings may provide the means to further understand the molecular mechanisms of surgery on resulting tumor biology, potentially facilitating the development of novel therapeutic agents aimed at resolving MB surgery-induced tumor proliferation.

MB disseminates into the CSF and spreads to other regions within the CNS. This highly migratory phenotype presents numerous drug delivery obstacles, which must be considered in potential therapeutic approaches. NSCs may be an effective model system for MB therapy as they have inherent tumor-tropic abilities [19, 20]. In this study, we observed NSC migration toward MB cancer cells *in vitro* and *in vivo*. NSCs administered in the contralateral cerebellar hemisphere migrated towards and co-localized with established MB tumors. Consistent with other reports [16], this suggests that NSCs display tropism towards MB. The ability of NSCs to migrate long distances and populate distant foci is likely dependent on their ability to persist in the brain. We found that NSCs persist for one week after prodrug injection, which ensures eventual clearing of the NSC population, but provides a limited window in which NSCs can migrate to MB cancer cells. This limited persistence could reduce the ability of NSCs to reach distant or metastatic sites, particularly as this approach is scaled to human patients. In the current study, we only explored GCV initiation at 24 h after NSC implantation, but prodrug administration could be delayed to provide more time for NSC homing. Currently, we are exploring this and other strategies to increase the time therapeutic stem cells have to reach tumor cells in distant locations.

NSCs genetically engineered to express cytotoxic agents are known to significantly diminish tumor burden and extend survival in orthotopic mouse models of malignant gliomas [9, 11, 13, 14, 37, 38], as well as post-surgical glioblastoma [15, 18, 20, 25, 29, 39, 40]. However, NSCs have not been explored in an orthotopic MB resection model. In this study, we showed that intra-cavity cytotoxic NSCs could limit re-growth of MB tumors after surgical resection. NSC therapy improved survival in two molecularly distinct MB tumor models (D283 and Daoy). NSC^tk^ therapy improved survival of mice with Daoy tumors more than two-fold compared to control mice, representing treatment of the Shh subgroup of MB. The D283 model represents tumors of the group 3 subgroup, which frequently metastasize and lead to the poorest prognosis of all MB subgroups. Implantation of hp-iNSC^tk^ into the post-surgical D283 resection cavity suppressed tumor growth and extended median survival in that aggressive group 3 tumor model. The ability of NSCs to control recurrence of two genetically distinct tumor models demonstrates the potential of NSC therapy in post-operative disease management of both intermediate and aggressive clinical phenotypes.

To date, the majority of preclinical and clinical studies have focused on allogeneic NSCs, but autologous cells hold potential to evade immune rejection and where the prolonged persistence could enhance treatment durability [41, 42]. Advances in cellular reprograming have led to the ability to reprogram a patient’s somatic cells into autologous cell therapies for personalized medicine [43]. Transdifferentiation (TD), the latest advancement in the reprogramming field, has allowed for quick and efficient cell type transitions [44]. TD generates terminally differentiated cells without passing through an undifferentiated pluripotent state, reducing the time required for generation of therapeutic cells and reducing the risk of *in vivo* teratoma formation. The safety benefit of TD-derived NSCs makes this therapy an attractive method for cell-based MB treatment. Through TD, skin fibroblasts can be used to generate hp-iNSCs in a matter of days [32, 45]. Additionally, fibroblasts obtained from young individuals are more readily reprogrammed [46, 47], suggesting this approach may be well suited for pediatric cancers such as MB. This is the first report of cell-based therapeutic efficacy in a cerebellar resection/recurrence tumor model. Our data demonstrated cytotoxic iNSCs homed to MB cells *in vitro*, suppressed tumor growth, and extended median survival in two genetically distinct MB resection/recurrence mouse models. Though stem cell therapy was effective against post-surgical MB, tumors eventually re-developed and led to death. Future studies on multimodal hp-iNSC therapy may provide an effective method to prolong treatment durability and eliminate therapy-resistant cell populations.

One of the limitations of our study is the use of established MB cell lines. The DAOY and D283 lines are commonly used and allowed us to complete our initial proof-of-concept testing. Studies have shown that low-passage primary patient-derived (PDX) cells lines or fresh tumor biopsy samples can more accurately recapitulate aspects of the clinical disease[48]. Future studies are planned to test the migration and killing of NSC drug carriers against these cells types. This will further enhance the clinical relevancy of our findings.

In conclusion, our study outlines the development of a novel MB resection model and the first demonstration that intra-cavity NSC/iNSC therapy is suppresses re-growth of post-operative MB. Development of the accurate MB resection/recurrence model presented in this study is vital to the understanding of recurrent MB disease and the subsequent advancement of recurrent MB therapies. This model mimics the aggressiveness of recurrent MB observed in the clinical setting, as resected tumors grew rapidly, spread through the cerebrum, and disseminated through the CSF. Utilizing this accurate pre-clinical model, we observed the ability of both brain-derived NSCs and hp-iNSCs derived from human pediatric fibroblasts to limit post-surgical MB tumor growth. This tumor model, coupled with the effectiveness of cytotoxic NSCs in the recurrent MB setting, provides a foundation for NSC-based therapies in the treatment of pediatric MB.

## Supporting information

S1 Checklist (PDF)

S2 Checklist (DOCX)

S1 Fig Representative images showing the migration of NSCs and hp-iNSC in control wells. (TIF)

S1 Supplemental methods (DOC)
