# Supplementary figures and images for: Intra-cavity stem cell therapy inhibits tumor progression in a novel murine model of medulloblastoma surgical resection
Source: PLoS One. 2018 Jul 10;13(7):e0198596. doi: 10.1371/journal.pone.0198596 (PMC6038981; doi:10.1371/journal.pone.0198596)

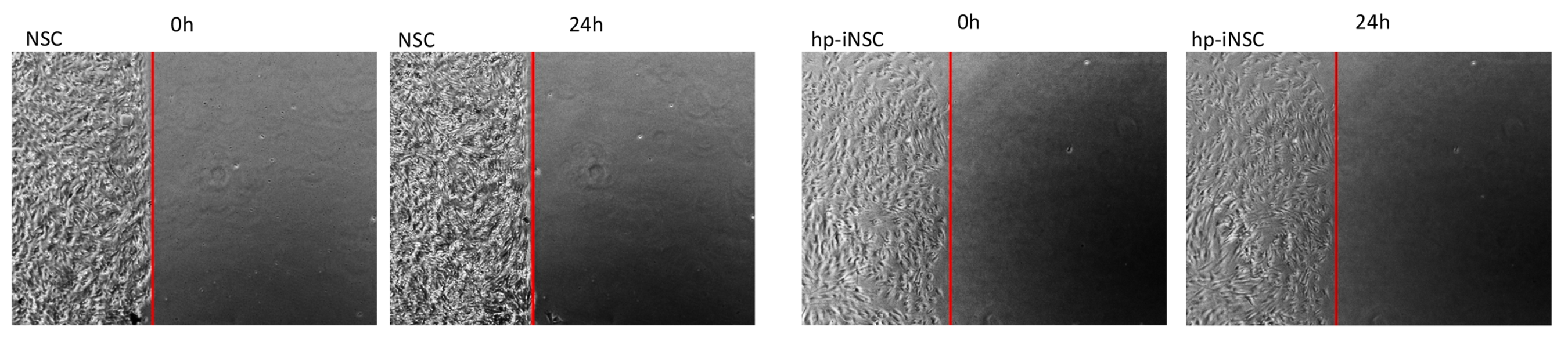

Supplement: S1 Fig — (TIF) [file pone.0198596.s003.tif]
