# Supplementary material for: Intra-cavity stem cell therapy inhibits tumor progression in a novel murine model of medulloblastoma surgical resection
Source: PLoS One. 2018 Jul 10;13(7):e0198596. doi: 10.1371/journal.pone.0198596 (PMC6038981; doi:10.1371/journal.pone.0198596)
Supplement: S1 Supplemental methods — (DOC) [file pone.0198596.s004.doc]

**Supplemental Methods**

**Cell culture**

Daoy (ATCC HTB-186), D283 (ATCC HTB-185), C17.2 (NSC, sigma) cell lines were cultured at 37°C and 5% CO_2_ in complete media consisting of DMEM (Gibco, Grand Island, NY) supplemented with 10% heat-inactivated fetal bovine serum (Sigma, St. Louis, MO), penicillin (100 µg/mL, Sigma), and streptomycin (100 µg/mL, Sigma), as previously described.[1] Lentiviral vectors (LV) engineered to express GFP-Firefly luciferase (LV-GFP-FLuc), mcherry-Firefly luciferase (LV-mcF), or RFP-Thymidine Kinase (LV-TK-RFP) were packaged in 293T/17 cells as previously described.[2] Daoy and D283 cells were transduced with LV-GFP-FLuc (TRP-GFPFL) in culture medium containing polybrene (6 µg/ml, Sigma). GFP expression was examined by fluorescence microscopy using an Olympus IX2 microscope equipped with a DP72 camera (Center Valley, PA).

**Human pediatric induced neural stem cell generation**

Human pediatric iNSCs (hp-iNSC) were generated using Sox2 under feeder-free conditions as described previously.[3, 4] Briefly, human pediatric fibroblasts (NHF1-hTERT),[5] a gift from William Kauffman, were transduced with a Dox-inducible Sox2 lentiviral vector in media containing polybrene and cultured in STEMdiff Neural Induction Medium (StemCell Technologies, Vancouver, Canada) containing doxycycline (10 μg/ml, Sigma). A week after transdifferentiation, these cells were transduced with LV-TK-RFP (hp-iNSC^tk^) in culture medium containing polybrene and visualized as described above.

**Bioluminescence imaging, *in vitro* and *in vivo***

To determine the correlation between the number of transduced cells and bioluminescence signal intensity, Daoy-GFP-FLuc cells were seeded in 96-well plates at varying concentrations (2x10^3^, 4x10^3^, 6x10^3^, 8x10^3^, 1x10^4^; N = 6 each). Luciferase activity was measured with an IVIS Kinetic 5 minutes after adding luciferase substrate (1 µg/mL). Data (mean ± SEM) were fit using linear regression.

Pre-resection tumor volumes were monitored by BLI using an IVIS Kinetic on day 0, 14, 21, 28, 35, 42, and 49 following tumor cell implantation. Surgery was performed on day 49 and tumor recurrence was monitored on post-operative day 0, 1, 3, and 5 (days 49, 50, 52, and 54 from cell injection). Images were captured 10 minutes following intraperitoneal injection of D-luciferin (1 mg per animal in 100 µL of PBS) using a 30 second or one minute acquisition time. Bioluminescence flux (photons/sec/cm^2^) was quantified using PerkinElmer LivingImage software and expressed relative to mean flux of initial (day 0 pre-resection).

**Orthotopic xenografts and fluorescence-guided microsurgery**

Three to four week old female nude mice (Charles River Laboratories, Wilmington, MA) were immobilized on a stereotactic frame (Stoelting, Wood Dale, IL) and a 2 mm circular portion of skull was removed three days prior to cell injection. Daoy-GFPFL or D283-GFPFL cells were harvested at 80% confluency, washed and resuspended in phosphate buffered saline (PBS). 1 × 10^5^ or 1 × 10^6^ cells per mouse (2 µL) were injected stereotactically in the right cerebellum at coordinates 4 mm lateral to the midline, 2 mm posterior to the lamboidal suture, and 0.5 mm deep.

Mice bearing established Daoy-GFPFL or D283-GFPFL xenografts were randomized to receive surgery (N=12, Daoy; N=12, D283). Mice were anesthetized and placed on a stereotaxic frame under an Olympus MVX10 microscope for intraoperative fluorescence visualization. After a scalp excision along the midline to visualize the underlying GFP^+^ tumor, an aspirator was used to debulk tumor volumes as previously described.[2] Bioluminescent imaging was used to measure tumor burden pre-resection immediately post-resection, and at serial time points post-surgery to determine growth rate of recurrent tumors. The maximum size that tumors were allowed to grow was 3 mm in diameter. Animal studies were approved by the University of North Carolina Institutional Animal Care and Use Committee.

**Histopathology and immunohistochemistry**

Brains harvested from tumor-bearing mice were immersed and fixed in 10% neutral buffered formalin overnight, and stored in 70% ethanol prior to paraffin embedding. Formalin-fixed, paraffin embedded brains were cut in the sagittal plane on a rotary microtome in serial 4 μm sections, placed on glass slides, and either remained unstained or stained with hematoxylin and eosin (H&E) on a Leica Microsystems Autostainer XL (Buffalo Grove, IL) in the UNC Translational Pathology Laboratory. Histopathological analysis was performed by CRM. Photomicrographs were taken on an Olympus MVX10 or Olympus BX41 microscope equipped with a DP71 digital camera. Unstained fluorescent brain sections were cover-slipped using Fluoro-Gel (Electron Microscopy Sciences, Hatfield, PA). Images were captured using an Olympus IX2 microscope equipped with a DP72 camera or an Olympus Fluoview Confocal FV 1200 inverted microscope.

**NSC migration to human medulloblastoma *in vitro* and *in vivo***

NSC-mcF or hp-iNSC-mcF (1x10^4^) cells were seeded in the presence or absence of Daoy-GFPFL or D283-GFPFL (1x10^4^) cells. Cell populations were separately seeded into adjacent wells located 0.5 mm apart in two-chamber culture-inserts (Ibidi, Verona, WI), placed in glass bottom microwell dishes (MatTek, Ashland, MA), and incubated overnight in complete media. Cells were placed in a VivaView incubator microscope (Olympus) and imaged at 10x every 20 minutes for 24 h. ImageJ was used to generate videos and perform single cell analysis to determine distance and velocity of migration of NSCs. Data (means ± SEM) were compared using Student’s *t*-tests.

To assess the *in vivo* migratory capability, NSCs (3x10^6^) were injected into the contralateral cerebellar hemisphere from Daoy xenografts that established for 7 days. Mice brains were harvested three weeks after tumor cell implantation, formalin-fixed, sectioned coronally and mounted as described above. Fluorescent images were captured throughout the tumor mass and analyzed using ImageJ. Images were taken with an Olympus IX2 microscope equipped with a DP72.

***In vitro* and *in vivo* therapy with survival analysis**

To assess the *in vitro* therapeutic efficacy of thymidine kinase-expressing stem cell therapy, NSC^tk^ (0, 5x10^2^, 1x10^3^, 5x10^3^, 1x10^4^, 2x10^4^; N = 8 each) or hp-iNSC^tk^ (0, 5x10^2^, 1x10^3^, 5x10^3^, 1x10^4^, 2x10^4^; N = 10 each) were cultured with Daoy-GFPFL (1x10^4^ cells) or D283-GFPFL (1x10^4^ cells). Therapeutic response was determined 48 hours after Ganciclovir (GCV) administration (100mg/kg daily via i.p. injection). Bioluminescent images were captured 5 minutes following Luciferin injection using a 10 second exposure. Images were processed and analyzed using LivingImage software.

To assess the persistence of NSC^TK^ in the surgical cavity, the cells were additionally engineered with the mC-F and seeded into the post-operative tumor cavity.[6] Twenty-four hours following surgery, mice received systemic administration of the prodrug, GCV or saline. Bioluminescent images were taken to measure the levels of residual NSC within the cavity. Images were captured 10 minutes following Luciferin injection using a 1 minute exposure and processed and analyzed using LivingImage software.

To assess the *in vivo* therapeutic efficacy of thymidine kinase-expressing stem cell therapy, hp-iNSC^tk^ or NSC^tk^ were implanted into the surgical cavity following microsurgical tumor debulking as previously described.[6] Twenty-four hours following surgery, mice received systemic administration of the prodrug, GCV or saline. Bioluminescent images were taken to measure tumor burden and evaluate therapeutic response. Images were captured 10 minutes following Luciferin injection using a 1 minute exposure and processed and analyzed using LivingImage software. Mice were monitored for neurological symptoms, sacrificed upon their development, and data analyzed as previously described.[2] Survival was evaluated by the Kaplan-Meier method and the effects of treatment compared by log-rank tests.

**Study approval**

All experimental protocols were approved by the Animal Care and Use Committees at The University of North Carolina at Chapel Hill, and care of the mice was in accordance with the standards set forth by the National Institutes of Health Guide for the Care and Use of Laboratory Animals, USDA regulations, and the American Veterinary Medical Association. Nude mice were purchased from an in-house colony. To ensure humane endpoints were followed in all procedures, the endpoint of the study for each animal is the time point at which it is sacrificed, or any time sooner when the animal is perceived to be suffering as evidenced by loss of feeding and drinking behavior, excessive quiet behavior, lack of grooming or seizures. Animals were monitored daily allowing for sacrifice or euthanasia to occur within 24 hrs of symptom onset. Weight loss was evaluated via body condition scoring. A maximum threshold of BC2 was in place at which point animals would be evaluated for veterinary care procedures. Additionally, if mice exhibited BC1 would immediately be euthanized. Any animal that died prematurely would be eliminated from the study to avoid altering data. Our approved animal protocol has allotted a 10% failure rate that leads to euthanasia of mice due to surgically or tumor-related complications. We did observe a significant number of failures in the current study. Inhaled isoflurane, 4-5% for induction and 2-3% for maintenance, was used as anesthesia. Cervical dislocation was used for euthanasia. To minimize pain and distress, bupivacaine was administered at the incision site, and animals received carprofen for 3 days post-operatively. Animals were housed in DCM facilities and were provided routine care by DCM staff. Mice were grouped in batches of 5 in sterile cages with HEPA-filtered air supply and autoclaved food. Cages included plastic housing structures and bedding material for environmental enrichment. Room lighting was cycled light/dark every 12 hrs. Animal numbers are detailed in the figure legends.

**Statistical analysis**

Data were analyzed with GraphPad Prism 6 (San Diego, CA). All comparisons were considered significant at P<0.05.

**References**

1. Okolie O, Bago JR, Schmid RS, Irvin DM, Bash RE, Miller CR, et al. Reactive astrocytes potentiate tumor aggressiveness in a murine glioma resection and recurrence model. Neuro-oncology. 2016. doi: 10.1093/neuonc/now117. PubMed PMID: 27298311.

2. Hingtgen S, Figueiredo JL, Farrar C, Duebgen M, Martinez-Quintanilla J, Bhere D, et al. Real-time multi-modality imaging of glioblastoma tumor resection and recurrence. Journal of neuro-oncology. 2013;111(2):153-61. Epub 2012/12/18. doi: 10.1007/s11060-012-1008-z. PubMed PMID: 23242736; PubMed Central PMCID: PMC3548430.

3. Bago JR, Okolie O, Dumitru R, Ewend MG, Parker JS, Werff RV, et al. Tumor-homing cytotoxic human induced neural stem cells for cancer therapy. Science translational medicine. 2017;9(375). doi: 10.1126/scitranslmed.aah6510. PubMed PMID: 28148846.

4. Bago JR, Alfonso-Pecchio A, Okolie O, Dumitru R, Rinkenbaugh A, Baldwin AS, et al. Therapeutically engineered induced neural stem cells are tumour-homing and inhibit progression of glioblastoma. Nature communications. 2016;7:10593. doi: 10.1038/ncomms10593. PubMed PMID: 26830441.

5. Boyer JC, Kaufmann WK, Cordeiro-Stone M. Role of postreplication repair in transformation of human fibroblasts to anchorage independence. Cancer Res. 1991;51(11):2960-4. Epub 1991/06/01. PubMed PMID: 1903328.

6. Bago JR, Pegna GJ, Okolie O, Hingtgen SD. Fibrin matrices enhance the transplant and efficacy of cytotoxic stem cell therapy for post-surgical cancer. Biomaterials. 2016;84:42-53. doi: 10.1016/j.biomaterials.2016.01.007. PubMed PMID: 26803410.
